# Uranium Interaction with Two Multi-Resistant Environmental Bacteria: *Cupriavidus metallidurans* CH34 and *Rhodopseudomonas palustris*

**DOI:** 10.1371/journal.pone.0051783

**Published:** 2012-12-12

**Authors:** Isabelle Llorens, Guillaume Untereiner, Danielle Jaillard, Barbara Gouget, Virginie Chapon, Marie Carriere

**Affiliations:** 1 ESRF-CRG-FAME, Polygone Scientifique Louis Néel, Grenoble, France; 2 Laboratoire Nanostructure et Rayonnement Synchrotron, CEA/DSM/INAC, Grenoble, France; 3 Laboratoire Structure et Dynamique par Résonance Magnétique, CEA/DSM/IRAMIS/SIS2M, UMR 3299 CEA-CNRS, Gif-sur-Yvette, France; 4 Centre Commun de Microscopie Electronique, UMR 8195 CNRS-Université Paris-Sud, Orsay, France; 5 ANSES, Direction scientifique des Laboratoires, Maisons-Alfort, France; 6 Laboratoire Interaction Protéine-Metal, CEA/DSV/IBEB, Saint-Paul-lez-Durance, France; 7 CNRS, UMR 7265, Saint-Paul-lez-Durance, France; 8 Université Aix-Marseille, Saint-Paul-lez-Durance, France; 9 Laboratoire Lésions des Acides Nucléiques, CEA/DSM/INAC/SCIB, UMR E3 CEA-Université Joseph Fourier, Grenoble, France; RMIT University, Australia

## Abstract

Depending on speciation, U environmental contamination may be spread through the environment or inversely restrained to a limited area. Induction of U precipitation via biogenic or non-biogenic processes would reduce the dissemination of U contamination. To this aim U oxidation/reduction processes triggered by bacteria are presently intensively studied. Using X-ray absorption analysis, we describe in the present article the ability of *Cupriavidus metallidurans* CH34 and *Rhodopseudomonas palustris*, highly resistant to a variety of metals and metalloids or to organic pollutants, to withstand high concentrations of U and to immobilize it either through biosorption or through reduction to non-uraninite U(IV)-phosphate or U(IV)-carboxylate compounds. These bacterial strains are thus good candidates for U bioremediation strategies, particularly in the context of multi-pollutant or mixed-waste contaminations.

## Introduction

Weathering of uranium (U)-containing natural minerals, industrial activities and the extensive use of phosphate fertilizers has resulted in a widespread U contamination of the environment, as reviewed by Hu et al. [1]. In Saxony, Germany, U mining and milling resulted in the release of millions of cubic meters of residues that still contain substantial amounts of U. Water may then transport U out of these solid waste; measurements showed that seepage waters, mine waters and tailing water contained up to 25 µM of U in this region [2]. Nuclear waste is also stored in the former U.S. nuclear weapon production sites such as Oak Ridge. These storage areas have created large contaminated sites where U concentration in sediments is up to 800 mg/kg, and pH and ionic strength in these areas have been described as being extreme [3], [4]. Intense research is focused on the decontamination of these sites, particularly by using bacteria or plants, i.e. bio- or phyto-remediation.

In surface soils and water, U is mainly present as U(VI) due to oxic conditions; and complexed to a variety of ligands such as (bi)carbonate, citrate or phosphate [5]. U(VI)-carbonate and U(VI)-citrate are highly mobile in the environment whereas complexation of U(VI) with phosphate leads to its immobilization. Under anoxic conditions, U is generally in the reduced, non-mobile U(IV) form [5]. A strategy to avoid dissemination of U in the environment consists in inducing its precipitation via biogenic or non-biogenic processes. To this aim U oxidation/reduction processes triggered by bacteria is presently intensively studied. As reviewed in 2008 by Merroun et al. [6], bacterial interactions with U include redox transformation, which are dynamic processes: U is reduced by some bacteria and re-oxidized by others. More than 25 phylogenetically different bacterial species are potential U reducing species [6], [7]. Most of these species reduce U to U(IV)-oxide minerals such as uraninite (UO_2_). Recent articles describe bacteria-mediated U(VI) reduction as non-uraninite U(IV) minerals. First, Khijniak et al. [8] reported U(VI)-phosphate reduction as ningyoite (CaU(PO_4_)_2_.H_2_O) by *Thermoterrabacterium ferrireducens*. Various Gram positive and negative bacteria, including five strains of *Desulfitobacterium* [9], [10], *Anaeromyxobacter dehalogenans* 2CP-C [9], *Shewanella putrefaciens* CN32 [9], *Shewanella oneidensis* MR-1 [11], *Desulfotomaculum reducens* MI-1 [11], *Clostridium* sp. [12] and *Clostridium acetobutylicum* [11] were described as reducing U(VI) not only to uraninite but also to U(IV)-carbonate or U(IV)-phosphate (ningyoite, U_2_O(PO_4_)_2_, U_2_(PO_4_)(P_3_O_10_)) minerals. The resulting mineral depends both on the involved bacteria and on the physicochemical conditions in exposure medium [11]. Bacterial interaction with U also includes complexation, by means of biosorption (ad- or absorption of U on bacterial surface) or intracellular accumulation [6]. Major sites of U sorption on bacterial cell walls are carboxylated and phosphorylated groups which are, depending on the wall structure of the considered bacteria, contained in peptidoglycans, secondary polymers (teichoic acids, teichuronic acids…), lipopolysaccharides, carbohydrate polymers constituting capsules, S-layer sheets or exopolysaccharides (for review see [6]).

Most of the U-contaminated areas are co-contaminated with other metals, radionuclides or pollutants such as organic compounds. Only indigenous bacteria or multi-resistant bacteria can be used in a perspective of bioremediation of these multi-polluted areas or mixed wastes. The objective of this study was to evaluate the potency of two highly resistant environmental bacteria to accumulate and immobilize U through U(VI)-phosphate precipitation or U(VI) reduction to U(IV), as well as to resist to high concentrations of U. Our aim was to compare U sequestration by either live or dead bacteria and at neutral and acidic pH; consequently we performed U exposure at pH 7 but also at pH 1. Our aim was to use these bacterial strains in conditions which were as close as possible to their classical laboratory culture conditions, in order to ensure that they kept all their specific resistance capacities. We thus did not add any electron donor or electron acceptor to exposure medium. *Cupriavidus metallidurans* CH34 was chosen for high resistance to a variety of metals, triggered either by efficient efflux pump or by cytoplasmic reduction/precipitation of toxic metals or metalloïds as non-toxic clusters [13], [14], [15]. *Rhodopseudomonas palustris* was chosen for its ability to survive in environments highly polluted by aromatic compounds [16].

## Experiments

### Cultures and media

Bacterial growth was followed by measurement of absorbance at 600 nm.

*C. metallidurans* CH34 was cultured aerobically at 29°C, in a rotary shaker, in Tris Salt Medium, pH 7.5. To avoid U precipitation, Tris buffer was replaced by 50 mM of sodium citrate in exposure medium; this medium was termed Citrate Salt Medium (CSM). *R. palustris* was grown anaerobically at 30°C in Hutner medium (20 mM Na_2_HPO_4_, 12 mM acetate, 20 mM malate, 8 mM glutamate, 16 µM nicotinic acid, 1 mM nitriloacetic acid, 15 µM 4-aminobenzoic acid, 7 µM EDTA, 3 µM thiamine, 164 nM biotine, 1 g/L yeast extract, pH 7) purged with O_2_-free N_2._ For U exposure, phosphate concentration was reduced to 1 mM (modified Hutner medium).

When bacterial cells reached the required optical density, 1 mM soluble U(VI) from an aqueous 30 mM uranyl-acetate stock was added to exposure medium.

### Uranium quantification

U content was measured either by ICP-MS in bacteria harvested by centrifugation or by UV-visible spectrometry in exposure media and post-exposure supernatants. Spectrophotometric determination of U was adapted from Texeira et al. (Texeira et al., 1999): samples were incubated in a reaction solution (V/V) composed of 38% reaction buffer (0.2 M of triethanolamine, pH 6.5), 38% complexing solution (50 g/L CyDTA, 5 g/L NaF, 130 g/L sulfosalicylic acid, pH 6.5), 8% of 0.04% 2-(2-Thiazolylazo)-p-Cresol solution, 8% of 50 mM N-acethyl-N,N,N-trimethylammonium bromide and 8% of 150 mM triton X-100. Absorbance at 588 nm was measured and U concentration was deduced from a calibration curve. For ICP-MS measurements, bacteria were lysed for 2 h at room temperature in Cell Lytic B (Sigma-Aldrich), then overnight in 20% SDS at 60°C. Samples were diluted in 2% HNO_3_ (v/v) (Normatom quality grade, VWR) and analyzed on an X7 Series quadrupole ICP-MS (Thermo Electron Corporation, France). Calibration curves were obtained from U standard solutions prepared with elemental SPEX CertiPrep standard (Jobin Yvon, France). ^9^Be, ^103^Rh and ^186^Re were used as internal standards (1 µg/L). SRM 1640 certified solution (NIST, USA) was analyzed at the beginning of each experimental batch to control the quality of analyses.

### Transmission electron microscopy observations

Bacteria were harvested by centrifugation, fixed at room temperature in 3% glutaraldehyde (vol./vol.) and 1% paraformaldehyde (vol./vol.) prepared in 0.1 M cacodylate buffer (pH 7.4), then post-fixed in 2% OsO_4_ (v/v) at 4°C. They were dehydrated in graded concentrations of ethanol and embedded in Epon resin. Ultra-thin sections (70 nm) were deposited on copper grids and observed on a CM12 Philips TEM operated at 80 kV.

### U speciation by X-ray absorption spectroscopy (XAS)

Bacteria were harvested by centrifugation, rinsed with ultrapure water and freeze-dried at −10°C under a vacuum of 0.37 mbar [17]. Standard samples were prepared and analyzed at room temperature as previously described [18]. XAS spectrum of meta-autunite was kindly provided by M. Merroun. U L_III_-edge XAS spectra, including both X-ray Absorption Near Edge Spectroscopy (XANES) and Extended X-Ray Absorption Fine Structure (EXAFS), were recorded on CRG-FAME (BM30B) beamline [19] at the European Synchrotron Radiation Facility storage ring (ESRF, France), operated in 4 bunches mode (4×10 mA) at 6 GeV. The monochromator was a Si(220) nitrogen-cooled double crystal [20] placed between 2 parabolic Rh-coated mirrors for harmonic rejection. Its energy was calibrated with a zirconium metallic foil by defining the first inflexion point at 17.998 keV. The beam size on the sample was 300×200 µm^2^ (FWHM, HxV). Fluorescence detection was achieved at the U Lα fluorescence line (13 615 eV) using a 30-element Ge solid state detector (Canberra).

EXAFS oscillations were isolated by removal of the pre-edge background, followed by μ_0_-removal via spline fitting using Athena, based on the IFEFFIT code [21]. Resulting EXAFS curves in the wavevector space (χ(k)) were k^3^-weighted. Spectra were Fourier transformed (FT) over the 1.1–12.3 Å^−1^ k-range using a Kaiser–Bessel apodization window, except for the *R. palustris*, pH 7 pellet (1–9 Å^−1^). They were refined by least-square minimization using Artemis program. Theoretical phases and amplitudes were calculated with FEFF6 code implemented in IFEFFIT programs suite based on Na_6_[U^IV^(CO_3_)_5_]12H_2_O [22], ningyoite CaU^IV^(PO_4_)_2_ [23], uranyl citrate [24]. Estimated structural parameters were N, coordination numbers; R, bond lengths; and σ^2^, Debye-Waller factors. During fitting procedure, the amplitude reduction factor (S_0_^2^) was at held constant at 1. N of the U−Oax single scattering (SS) path of uranyl species was fixed at 2 and all fits included the two-fold degenerated 4-legged multiple scattering (MS) path of the uranyl group Oax1 = U = Oax2. The bond length (R_MS_) and Debye-Waller factor (σ^2^_MS_) of the MS path were linked at twice the R_SS_ and σ^2^_SS_ values of the U−Oax SS path, respectively [25]. Shift in threshold energy (ΔE_0_) was varied as a global parameter.

## Results

### Impact of uranium on bacterial growth

In the classical culture conditions we used, a typical growth curve of *C. metallidurans* CH34 in TSM was composed of a short lag phase (∼2 h), then log phase (∼6 h) and then stationary phase.

**Figure 1:**
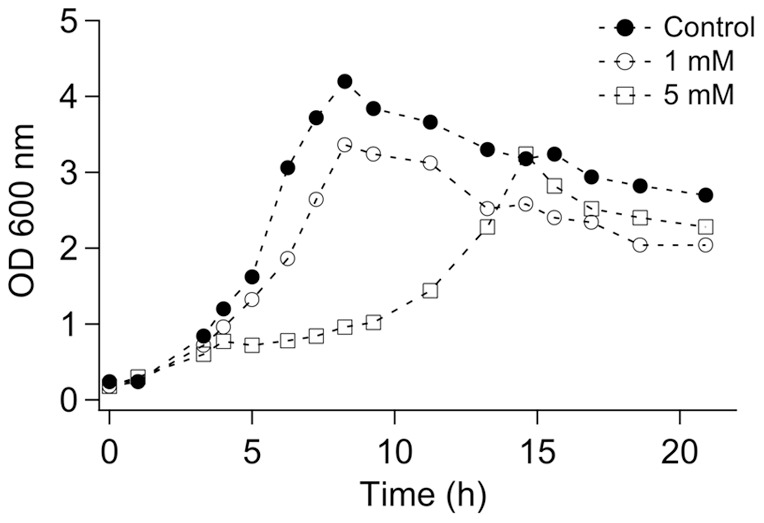
Bacterial growth upon U exposure. Typical growth curve of *C. metallidurans* CH34 in CSM medium (black circles) or CSM containing 1 mM of U (white circles) or 5 mM of U (white squares).

Growth in CSM induced a reduction of cell concentration in stationary phase: optical density reached 4.3 in CSM instead of 1.8 in TSM (Figure S1A). Exposure of bacteria to 1 mM of U in CSM reduced stationary phase cell concentration to 3.3 without affecting the lag phase while exposure to 5 mM of U decreased the stationary phase cell concentration and lengthened the lag phase (Figure 1). Maximal growth rate of *C. metallidurans* CH34 was reduced by 20–30% in CSM supplemented with 5–30 mM of U (Figure S1B). For U concentrations lower than 5 mM, growth rate reduction was not statistically significant. Exposure of *C. metallidurans* CH34 in CSM medium at pH 1, either containing U or not, was bactericidal, *i.e*. it immediately and irreversibly stopped bacterial growth.

The growth of *R. palustris* was slow in modified Hutner medium (1 mM of phosphate instead of the classically used 20 mM). After a lag phase of nearly 48 h, it reached the stationary phase after 4 days. Addition of 1 mM of U in modified Hutner medium did not modify the duration of growth phases, nor maximum growth rates, nor cell concentration in stationary phase (not shown).

### U accumulation

**Figure 2:**
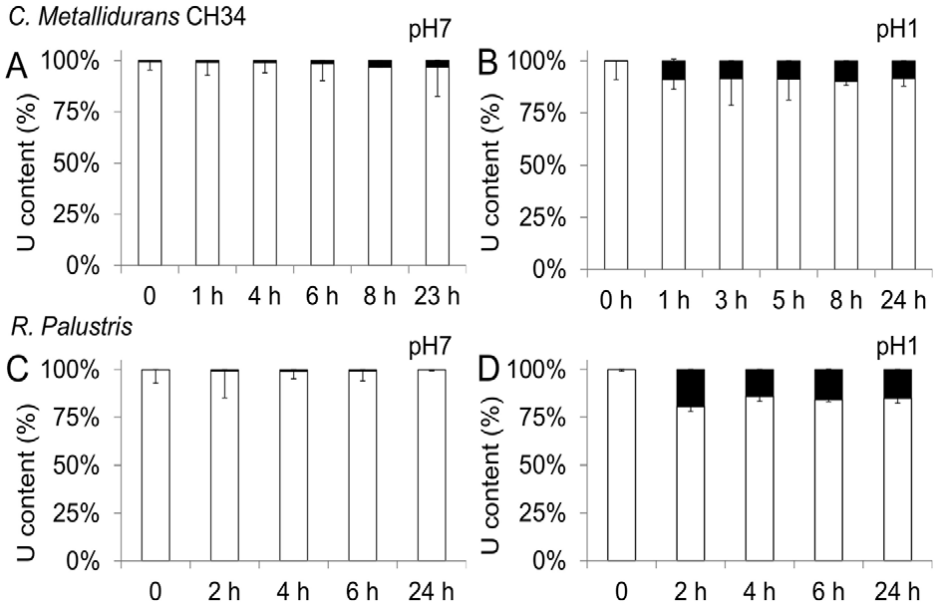
U accumulation in bacteria. U content was measured by ICP-MS in bacterial and by spectrophotometry in exposure media after exposure to 1 mM U of *C. metallidurans* CH34 at pH 7 (A), at pH 1 (B) or *R. palustris* at pH 7 (C) or pH 1 (D). White bar: U content in exposure supernatant; black bar: U content in bacterial pellet.

**Figure 3:**
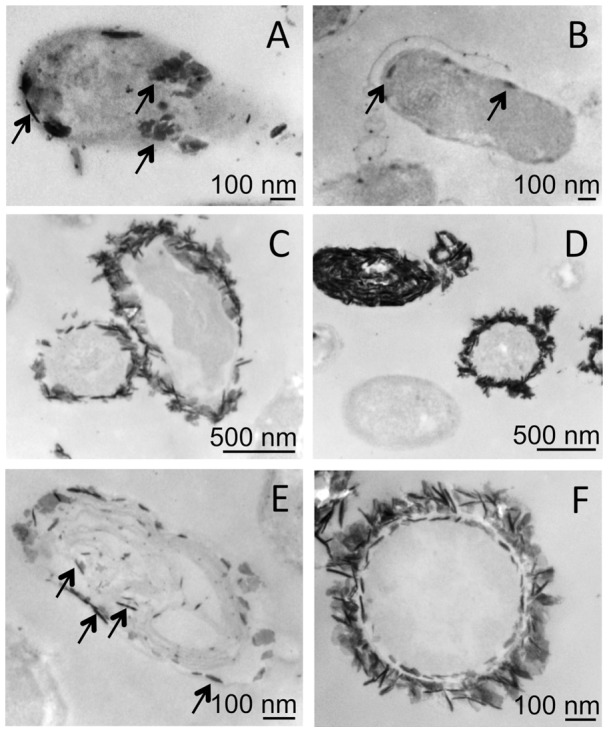
TEM observations of bacteria exposed to U. Exposure of *C. metallidurans* CH34 in CSM medium at pH 7 (A, 4 mM, 1 h) or pH 1 (B, 1 mM, 24 h), *R. palustris* in modified Hutner medium at pH 7 (C, D, 1 mM, 4 h) or pH 1 (E, F, 1 mM, 4 h).

When 1 mM of U was added in CSM pH 7 in the beginning of the log phase, U accumulation in *C. metallidurans* CH34, measured by ICP-MS, remained low (Figure 2A). TEM observation revealed the presence of electron-dense clusters in some bacteria sampled in the early stages of growth (Figure 3A, arrows), but this event was sporadic. At later growth times, no electron-dense cluster was observed. In order to compare U sequestration by either live or dead bacteria, and at neutral or acidic pH, we also performed U exposure at pH 1. *C. metallidurans* CH34 was grown in CSM medium, sampled in mid-exponential phase (optical density  = 2), centrifuged and exposed to 1 mM of U in CSM, pH 1. After 1–24 h of exposure, significant U content was measured in bacteria while U content in supernatants were reduced accordingly (Figure 2B). Scarce electron-dense deposits were observed on the surface of some bacteria (Figure 3B, arrows).

When exposed in modified Hutner medium, pH 7, U sequestration in *R. palustris* was very low (Figure 2C). After 4 h of exposure to 1 mM of U, TEM observation revealed the presence of two sub-populations of bacteria. A minor population was covered with electron dense deposits (Figure 3C–D) looking like U-phosphate precipitates as previously described in other species [18], [26], [27], [28]. Most of the bacteria looked like control bacteria, *i.e*. without any visible U deposits (Figure 3D, bottom left). When exposed to 1 mM of U, 15–20% of the initial U content was associated with the bacterial cells (Figure 2D). In this condition, a majority of cells present electron-dense deposits. These precipitates were either located in the cytoplasm or at the cell wall (Figure 3E, arrows). Sometimes, as shown in Figure 3F, a double layer of precipitates is clearly visible around a cell ghost; it might result from U deposition on bacterial inner and outer membrane.

### U speciation

**Figure 4:**
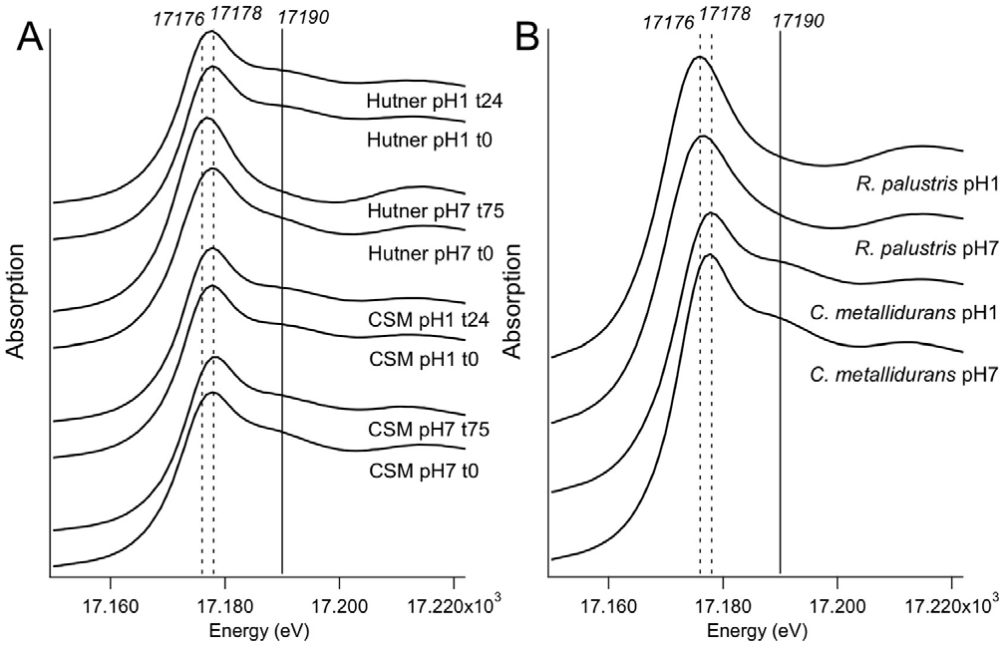
Normalized X-ray absorption spectra of culture media and bacteria. Bacterial media were analyzed at the U LIII-edge just after U amendment of after 24 h (pH 1) or 75 h (pH 7) of bacterial culture (A). At these time-points bacteria were sampled by centrifugation and pellets were analyzed (B).

U speciation was then evaluated by EXAFS in exposure media as well as in cells of *C. metallidurans* CH34 and *R. palustris* collected after 24 h (pH 1) or 75 h (pH 7) (Figure 4). Exposure media were analyzed either at the beginning of bacterial exposure (t0) and at the end.

In the XANES region, the energy position of the edge step was 17.178 keV in *C. metallidurans* pellets and exposure media at both pH 7 and pH 1 (Figure 4A–B). It was shifted to a lower energy, 17.176 keV, in *R. palustris* pellets at pH 7 and pH 1, suggesting that U was reduced (Figure 4B). In addition the shoulder after the white line, which signs the presence of O = U = O axial uranyl bond, specific to U(VI) species, was absent. This resonance has been attributed to multiple scattering paths within the linear uranyl cation, and lies ∼10 eV above the white line as described by Hudson et al. [29]. These evidences suggest that U(VI) was reduced to U(IV) in *R. palustris*. In Hutner medium at pH 1 and pH 7, t0, as well as in exposure medium at pH 1 after 24 h of exposure, the absorption edge was at 17.178 keV, indicating that U was in the U(VI) valence state (Figure 4A). In the exposure medium of *R. palustris* at pH 7 after 75 h of exposure, the energy of the edge step was also shifted, but only to 17.177 keV. The shoulder after the white line was flattened but still present. This indicates the presence of a mixture of U(VI) and U(IV).

**Figure 5:**
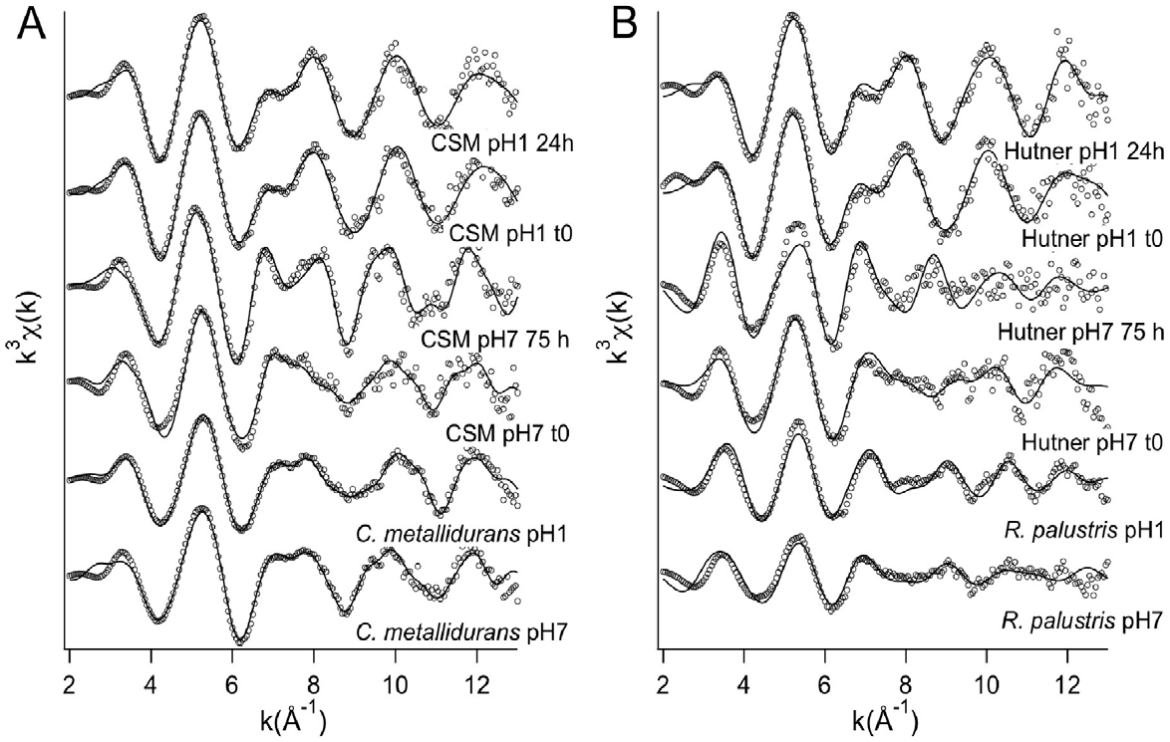
K^3^-weighted EXAFS oscillations extracted from X-ray absorption spectra of culture media and bacteria. Data (solid lines with circles) and best-fits (dashed lines).

U L_III_-edge EXAFS spectra are shown in Figure 5, best fit values are given in Table 1 and Table 2 for *C. metallidurans* CH34, Table 3 and Table 4 for *R. palustris*. The corresponding Fourier-transforms of EXAFS spectra are shown in Figure S2.

**Table 1:** Best fit values for *Cupriavidus metallidurans* CH34 at pH 7^a^.

Path	N	R(Å)	σ^2^ (10^−3^ Å^2^)	ΔE0	R-factor
*CSM t0*				10.5 (0.3)	0.009
U → Oax	2*	1.81 (0.01)	4 (1)		
U → Oeq2	5.6 (1.1)	2.38 (0.01)	11 (2)		
U → C	1.2 (0.7)	2.91 (0.05)	4 (5)		
U → U	0.6 (0.4)	4.32 (0.04)	3 (4)		
MS2 (U-Oeq-C)	4×NC	3.15 (0.09)	3*		
MS3 (U-C-C)	8.2 (4.3)	4.10 (0.04)	3*		
*CSM 75 h*				6.3 (0.7)	0.006
U → Oax	2*	1.80 (0.04)	2 (1)		
U → Oeq	5.8 (0.5)	2.41 (0.01)	7 (1)		
U → C	3.6 (0.6)	2.87 (0.01)	5 (2)		
U → Odist	NC	4.23 (0.02)	1*		
MS2 (U-Oeq-C)	4×NC	3.15 (0.03)	3*		
*C. metalliurans* pellet				7.2 (0.7)	0.006
U → Oax	2*	1.79 (0.01)	3 (1)		
U → Oeq1	4.2 (0.4)	2.34 (0.01)	6 (1)		
U → Oeq2	2.3 (0.4)	2.49 (0.01)	3 (3)		
U → P	0.3 (0.3)	3.59 (0.03)	3 (2)		
U → C	2.6 (0.7)	2.93 (0.01)	4*		
MS1 (U-Oeq1-P)	2*NP	3.81 (0.05)	σ^2^ (UP)		
MS2 (U-Oeq2-C)	4*NC	3.85 (0.05)	6*		

a So^2^ was fixed at 1 for all U L_III_ XAFS measurements. E_0_ (eV) was fitted as a global parameter. The number of Oax atoms was held constant at 2.0. * indicates that the value was held constant during fit procedure.

**Table 2:** Best fit values for *Cupriavidus metallidurans* CH34 at pH 1^a^.

Path	N	R(Å)	σ^2^ (10^−3^ Å^2^)	ΔE0	R-factor
*CSM t0*				5.4 (0.7)	0.007
U → Oax	2*	1.76 (0.01)	2 (1)		
U → Oeq	5.2 (0.5)	2.40 (0.01)	9 (1)		
U → C	2.1 (0.9)	2.94 (0.03)	5 (3)		
*CSM 24 h*				5.0 (0.1)	0.005
U → Oax	2*	1.76 (0.01)	3 (1)		
U → Oeq	5.0 (0.5)	2.40 (0.01)	9 (1)		
U → C	2.5 (0.8)	2.92 (0.02)	5 (2)		
*C. metallidurans* pellet				7.7 (0.3)	0.002
U → Oax	2*	1.77 (0.01)	4 (1)		
U → Oeq1	3.8 (0.3)	2.34 (0.01)	6 (1)		
U → Oeq2	1.1 (0.4)	2.49 (0.01)	5 (3)		
U → P	0.5 (0.2)	3.59 (0.03)	3 (2)		
U → C	1.7 (0.3)	2.94 (0.01)	2*		
MS1 (U-Oeq1-P)	2*NP	3.81 (0.05)	σ^2^ (UP)		
MS2 (U-Oeq2-C)	4*NC	3.85 (0.06)	4*		

a So^2^ was fixed at 1 for all U L_III_-edge XAFS measurements. E_0_ (eV) was fitted as a global parameter. The number of Oax atoms was held constant at 2.0. * indicates that the value was held constant during fit procedure.

**Table 3:** Best fit values for *Rhodopseudomonas palustris* at pH 7^a^.

Path	N	R(Å)	σ^2^ (10^−3^ Å^2^)	ΔE0	R-factor
*Hutner t0*				8.7 (1.2)	0.020
U → Oax	2*	1.80 (0.01)	6 (1)		
U → Oeq	6.5 (0.8)	2.34 (0.01)	11 (2)		
U → C	½ NOeq	2.95 (0.02)	5 (3)		
MS (U-Oeq-C)	½ NOeq	2.95*	3*		
*Hutner 75 h*				2.8 (0.9)	0.036
U → O	7.8 (0.8)	2.33 (0.01)	9 (1)		
U → P	7*	3.59 (0.03)	15*		
*R. palustris* pellet				4.0 (1.0)	0.049
U → O1	3.4 (0.4)	2.26 (0.01)	6 (1)		
U → O2	2.4 (0.5)	2.42 (0.02)	6 (1)		
U → P	1.5 (0.9)	3.11 (0.03)	9 (4)		

a So^2^ was fixed at 1 for all U L_III_-edge XAFS measurements. E_0_ (eV) was fitted as a global parameter. The number of Oax atoms was held constant at 2.0. * indicates that the value was held constant during fit procedure.

**Table 4:** Best fit values for *Rhodopseudomonas palustris* at pH 1^a^.

Path	N	R(Å)	σ^2^ (10^−3^ Å^2^)	ΔE0	R-factor
*Hutner t0*				4.5 (0.9)	0.009
U → Oax	2*	1.76 (0.01)	3 (1)		
U → Oeq	4.8 (0.5)	2.40 (0.01)	7 (1)		
U → C	3.3 (1.1)	2.93 (0.02)	5 (3)		
*Hutner 24 h*				4.0 (1.1)	0.009
U → Oax	2*	1.76 (0.01)	2 (1)		
U → Oeq	4.2 (0.4)	2.39 (0.01)	7 (1)		
U → C	2.9 (0.1)	2.89 (0.56)	1 (1)		
*R. palustris* pellet				1.1 (0.7)	0.020
U → O	6.0 (0.4)	2.28 (0.01)	9 (1)		
U → Odist	2.9 (2.2)	4.19 (0.05)	6 (5)		
U → C	3.4 (0.8)	2.90 (0.02)	5 (1)		
MS (U-O-C)	2.4 (1.0)	4.27 (0.02)	7 (3)		

a So^2^ was fixed at 1 for all U L_III_-edge XAFS measurements. E_0_ (eV) was fitted as a global parameter. The number of Oax atoms was held constant at 2.0. * indicates that the value was held constant during fit procedure.

In *C. metallidurans* exposed to U at pH 7 and pH 1, the EXAFS spectra are consistent with U associated with a combination of carboxylate and phosphate groups bound to a uranyl moiety. At pH 7 the EXAFS spectrum is correctly fitted with a shell containing 0.3 atoms of P at a distance of 3.59 Å together with a shell of 2.6 atoms of C at 2.93 Å. The model includes two oxygen atoms at 1.79 Å, consistent with the parameters of the uranyl cation, and two shells of equatorial oxygen atoms at 2.34 and 2.49 Å. At pH 1, the best fit values are a shell of 0.5 atoms of P at 3.59 Å together with a shell of 1.7 atoms of C at 2.94 Å. The distance between U and the two axial oxygen atoms is 1.77 Å, and the two shells of equatorial oxygen atoms are at 2.34 and 2.49 Å (Table 2).

Regarding U speciation in *C. metallidurans* CH34 exposure medium (CSM) pH 7, t0, it is well fitted with a U(VI)-carboxylate model such as U(VI)-citrate, as described previously [24], [30]. The model includes 1.2 C atoms at 2.91 Å, a shell of two axial O atoms at 1.81 Å and a shell of 5.6 equatorial O atoms at 2.38 Å. The U-U contribution and U-Oeq-C and U-C-C multiple scattering paths significantly improve the quality of the fit (Table 1). After 75 h of exposure, the best fit of the spectrum recorded on exposure medium consist of 3.6 C atoms at 2.87 Å and 5.8 equatorial O atoms at 2.41 Å, and a significant contribution of distant oxygen atoms at 4.23 Å (Table 1). These parameters rather corresponds to those of a U(VI)-carbonate complex.

Finally in the exposure medium at pH 1, t0 and 75 h, the best fit values are consistent with a model of U(VI)-citrate at acidic pH [30], with ca. 5 equatorial oxygen atoms at 2.40 Å and 2–2.5 C atoms at 2.92–2.94 Å (Table 2).

Spectra recorded on *R. palustris* were noisier. Consequently R-factors, indicative of the fit quality, are more important than those obtained on *C. metallidurans* CH34. At pH 7 the best-fit model for *R. palustris* pellet consists of 1.5 P atoms at 3.11 Å and two shells of O equatorial atoms at 2.26 and 2.42 Å. No contribution of axial O is found, and the ΔE0 is lower than ΔE0 found in *C. metallidurans* CH34 best-fit models. This model is consistent with a U(IV)-phosphate compound [8], [9], [11]. The EXAFS spectrum of exposure medium at t0 fits with a U(VI)-carbonate or U(VI)-citrate compound. The best-fit model involves 6.5 equatorial O atoms at 2.34 Å and half this number of C atoms at 2.95 Å. After 75 h of exposure, the spectrum recorded on exposure medium is quite different and is noisy. It however clearly shows a U(IV) compound where U is bound to 7 P atoms at 3.59 Å and 7.8 O atoms at 2.33 Å, which is consistent with a U(IV)-phosphate. At pH 1, in exposure media the spectra are qualitatively similar to those obtained on *C. metallidurans* CH34 exposure media at pH 1. They are correctly fitted with a model of U(VI)-citrate. This model includes 4.8 or 4.2 Oeq at 2.40 or 2.39 Å, and 3.3 or 2.9 C atoms at 2.93 or 2.89 Å, at t0 and after 24 h of exposure, respectively (Table 4). At this acidic pH, the best-fit model for bacterial pellets consists of 6.0 equatorial O atoms and 3.4 C atoms. This model does not contain any contribution of axial O atoms, which is consistent with a U(IV)-carbonate species.

## Discussion

Uranium speciation modification upon interaction with bacteria is related to the valence state, i.e. U(VI) is reduced to U(IV)-also termed bioreduction- (for review, see [6] and [8], [9], [10], [11], [31], [32] for more recent studies) or inversely U(IV) is oxidized to U(VI). Alternatively it is related to U(VI) complexation with biogenic ligands-also termed biosorption-such as biomolecules containing phosphoryl or carboxyl groups [6], [25], [33], [34], [35]. In the present article we describe the ability of two bacteria, highly resistant to specific environmental pollution situations, to immobilize U. *C. metallidurans* CH34, resistant to a variety of metals and metalloids, immobilizes U(VI) by complexing it with phosphoryl- or carboxyl-containing biomolecules. It resists to up to 30 mM of U(VI) with only a slight decrease of growth rate. *R. palustris*, resistant to aromatic pollutants, immobilizes U(VI) by reducing it to U(IV)-phosphate or to U(IV)-carboxylate without any loss of viability. U sequestration in both bacteria in these conditions is low. However, considering that these bacteria are able to grow in polluted areas while other bacteria which would immobilize higher U concentrationwould not survive in such environments, *C. metallidurans* CH34 and *R. palustris* can be considered good candidates for bioremediation particularly for the remediation of U(VI)-contaminated effluents in a situation of mixed-waste pollution. For instance, large areas with subsurface contaminant plumes of U and other metals exist, particularly in the U.S. close to nuclear weapon production sites, but also in France, Germany and other European countries. Several remediation strategies are currently envisaged for these contaminated areas, and among them bioremediation by *in situ* biostimulation is envisaged in the U.S. Oak Ridge site [3]. These areas contain up to 800 mg U per kg of sediment, the soils are acidic, the ionic strength is high and these sites are co-contaminated with a variety of other chemical species and metals [3]. In these extreme conditions, few exogenous bacteria would be able to survive and reproduce. Assays aiming at the bioremediation of these sediments have been performed by in situ biostimulation [3] or in microcosms [4]. In the microcosm experiment, initial U concentration was about 12 µM in the liquid phase, which is much lower than the concentration used in our study. After 11 months of microcosm culture, U concentration was lower than 0.1 µM, i.e. bioremediation was very efficient. The authors describe the influence of this harsh environment on bacterial communities, and diversity is shown to be reduced after 5 months in the microcosm [4]. We also previously reported the presence of specific bacterial communities, which were very stable over time, in natural soils containing high concentrations of U [36]. One strategy to remediate such sites is to use indigenous bacteria by biostimulating their growth, *in situ*. These bacteria would then immobilize U and limit its diffusion to other compartments of the environment. However in this strategy one bacterial strain (or a series of bacterial strains) would be efficient to decontaminate only one polluted site, with its specific biotic and abiotic conditions. The approach described in the present article would provide an alternative strategy for the bioremediation of contaminated sites, giving the opportunity to apply a series of selected exogenous bacteria to several contaminated sites, with different biotic and abiotic conditions. We used a *C. metallidurans* CH34 exposure medium containing a high concentration of citrate. Note here that we used citrate in these laboratory experiments because it ensured the maintenance of U in a soluble form in bacteria exposure media. In an environmental remediation perspective it would be better not to use citrate, since it is described as a mobilizing agent for U [37]. The consequence of its application on U-containing soils would be U leaching while our aim is rather to immobilize U. Our experimental results confirm that in this medium U is complexed to a carboxylate compound at both pH 7 and pH 1, with coordination number and distances consistent with those of (VI)-citrate. Indeed at pH 7, the significant presence of a U-C-C multiple scattering path in the best-fit model, with a coordination distance of 4.10 Å, is consistent with a U(VI)-citrate binding rather than a U(VI)-carbonate or -acetate binding. At pH 1, the coordination number of U with equatorial oxygen atoms is around 5 as in U(VI)-citrate, while it is rather around 6 in U(VI)-acetate or U(VI)-carbonate.

In bacteria at the end of the exposure time, U is found as a mixture of U(VI)-phosphate and U(VI)-carboxylate. *C. metallidurans* CH34 is a Gram negative bacteria; its cell wall is composed of a phospholipid-, polysaccharide- and lipopolysaccharide (LPS)-rich outer membrane. LPS is rich in phosphoryl and carboxyl groups on which U(VI) may bind [6], [25], [34], [35]. Barkleit *et* *al*. recently characterized the interaction between U(VI) and LPS and showed that U(VI) was either complexed with phosphoryl groups only, or with a mixture of phosphoryl and carboxyl groups, depending on the U(VI)/LPS-functional groups molar ratio [33]. When LPS is present in an excess molar ratio, U(VI) is rather complexed with organic phosphate. When the system is composed of a nearly equimolar ratio of U(VI) to LPS functional groups then the U(VI) coordination sphere is composed of phosphate and carboxylate. Our conclusions are very similar: EXAFS spectra fit clearly shows the presence of two types of equatorial oxygen atoms, with coordination distances consistent with those of a monodentate phosphate binding (2.34 Å) together with a bidentate carboxylate binding (2.49 Å). The coordination distance between U and equatorial oxygen atoms from phosphate groups are quite long, consistent with the coordination of organic rather than inorganic phosphate [6], [33], [34]. Nevertheless direct interaction of U with P atoms is not clearly found since the coordination number of P is 0.3±0.3 at pH 7 and 0.5±0.2 at pH 1. As a conclusion, our experimental results strongly suggest that U is bound to *C. metallidurans* CH34 LPS layer at both pH 1 and pH 7, coordinated to both phosphate and carboxylate groups. Several hypotheses can be proposed to explain the higher ad-/absorption of uranium in *C. metallidurans* CH34 at pH 1, as compared to pH 7. First, at pH 7 bacteria are alive; the only phosphoryl- and carboxyl-groups to which U(VI) may bind are those exposed on the external surface of their cell wall. At pH 1 bacteria must be lysed, i.e. their cell wall is leaky, releasing other potential U(VI)-binding groups from the periplasm or the cytoplasm, as previously suggested [33]. Second, the bacterial surfaces may evolve in response to changing U exposure conditions [6]. Particularly their surface structures may be different at pH 1, as compared to pH 7. It is recognized that in laboratory experiments bacteria often lose their proteinaceous surface layer (S-layer) [38]. It is possible that at pH 1 *C. metallidurans* CH34 produces an S-layer that is absent at pH 7, or produces a different S-layer at pH 1 and at pH 7. It is also possible that, due to fixation of U on the S-layer, the turnover of bacterial surface is induced. Production of a new S-layer would then lead to the production of new U binding sites, which would then induce another round of U adsorption on bacterial surface. Another explanation would be the presence of different quantities of exopolysaccharides (EPS) at pH 1 as compared to pH 7; these polymers have been previously proved to bind U at low pH in *Acidithiobacillus ferrooxidans* [39].

At pH 1, U speciation in exposure medium remains the same throughout the exposure period, i.e. U(VI)-citrate. Conversely at pH 7 the best-fit model suggests that after 75 h of exposure U is coordinated to a carboxylate, but not citrate: in this spectrum the U-C-C multiple scattering path is not significant but coordination of U(VI) to a distant oxygen atom significantly improves the fit. Our hypothesis is that active *C. metallidurans* cells accumulate U(VI)-citrate, then this complex dissociates in the cytoplasm or in the periplasmic compartment and U(VI) recombines with another carboxylate produced through bacterial respiration. This newly formed U(VI)-carboxylate complex is then released in the exposure medium. This hypothesis would be another explanation of higher U accumulation at pH 1: since bacteria are not metabolically active at this pH, U(VI)-citrate cannot be recombined and released in exposure medium, thus remaining fixed on bacterial membrane residues.

While biosorption is the predominant interaction between U and *C. metallidurans* CH34, we demonstrate in the present article that *R. palustris* reduces U(VI) as U(IV)-phosphate or as U(IV)-carboxylate. U is initially present as U(VI)-carboxylate in exposure medium at pH 1 and pH 7. Hutner medium contains high concentrations of acetate, malate and glutamate. Malate and glutamate contain α-hydroxyl groups which may bridge uranyl ions [24], [40]. Moreover EXAFS spectra of U(VI)-citrate and -malate show strong similarities [24]. It is probable that U(VI)-glutamate EXAFS spectrum is also very similar, then it is unreasonable to consider that we would differentiate these compounds in exposure medium. We thus can only state that U is initially present as U(VI)-carboxylate in exposure medium. In bacteria, EXAFS spectra are noisy but clearly indicate that U(VI) has been reduced to non-uraninite U(IV). The first coordination sphere of U(IV) contains O atoms; the second coordination sphere contains P atoms at pH 7 and C atoms at pH 1. Coordination distances are consistent with those described by Boyanov et al. [9] and in the model recreated by Hennig *et* *al*. [22] and cited by Boyanov *et* *al*. However in our experiments the quality of data does not allow the addition of parameters which would describe several spheres of O and P. The coordination numbers are lower than those described by Hennig *et* *al*., but the quality of our data do not permit to perfectly fit the spectrum. Alternatively, our XANES spectra show the predominance of a U(IV) species but also the presence of a minor contribution of a U(VI) compound. The presence of this U(VI) compound contributes to the overall noise in the spectrum and introduces uncertainty in the fit.

Interestingly, Boyanov *et* *al*. describe that U(VI) is reduced to non-uraninite U(IV) by both Gram positive and Gram negative bacteria, but the resulting U(IV) compounds depend on the Gram coloration and composition of exposure medium. When Gram positive and Gram negative bacteria are exposed to U(VI) in a medium containing phosphate, U(VI) is reduced to U(IV)-phosphate. When the exposure medium lacks phosphate, Gram negative bacteria reduce U(VI) to uraninite while Gram positive bacteria reduce U(VI) to U(IV)-carboxylate with a minor contribution of uraninite. Reduction would occur directly at the surface of Gram negative bacteria thanks to the presence of reductases in the outer membrane, while it would occur in the exposure medium in Gram positive bacteria by the involvement of a soluble mediator [9]. *R. palustris* is Gram negative; the exposure medium we used contains phosphate. At pH 7 our results confirm the hypothesis of Boyanov *et* *al*.: U(VI) is reduced to U(IV)-phosphate. At pH 1 we rather observe a U(IV)-carboxylate. At pH 7 *R. palustris* cells are alive and would thus reduce U(VI) to U(IV)-phosphate on their surface *via* the outer membrane reductases. Conversely at pH 1 bacteria are not metabolically active: membrane reductases are not active and their cytoplasm and periplam are partly or totally released into the exposure medium. This may lead to the release of soluble reduction mediators which would favor U(IV) reduction as U(IV)-carboxylate rather than U(IV)-phosphate.

## Conclusions

We describe in the present article the ability of *C. metallidurans* CH34, highly resistant to metals and metalloids, as well as *R. palustris*, highly resistant to organic pollutants, to withstand and immobilize U. Thanks to their multi-resistance they could be used for the bioremediation of polluted areas containing both U and other pollutants. *C. metallidurans* CH34 cells, both at pH 7 (live) and at pH 1 (dead) immobilize U(VI)-citrate by biosorption as a mixture of U(VI)-phosphate and U(VI)-carboxylate. *R. palustris* immobilizes U(VI)-carboxylate by bioreduction either as U(IV)-phosphate (pH 7) or as U(IV)-carboxylate (pH 1). Future work will aim at enhancing their U sequestration capacity, by modulating exposure pH and the composition of exposure media in order to find optimal physico-chemical conditions for U accumulation and/or adsorption on bacterial wall.

## Supporting Information

Figure S1 **Growth of C. metallidurans CH34 in CSM vs. TSM medium.** C. metallidurans classical growth medium is TSM; in order to avoid U precipitation during exposure this growth medium was adapted, i.e. Tris buffer was replaced by citrate, leading to a growth medium denominated CSM.
(TIF)

Figure S2 **Fourier transforms of the EXAFS spectra recorded on exposure media and bacteria pellets.**
(TIF)
