# Supplementary figures and images for: Uranium Interaction with Two Multi-Resistant Environmental Bacteria: Cupriavidus metallidurans CH34 and Rhodopseudomonas palustris
Source: PLoS One. 2012 Dec 12;7(12):e51783. doi: 10.1371/journal.pone.0051783 (PMC3520905; doi:10.1371/journal.pone.0051783)

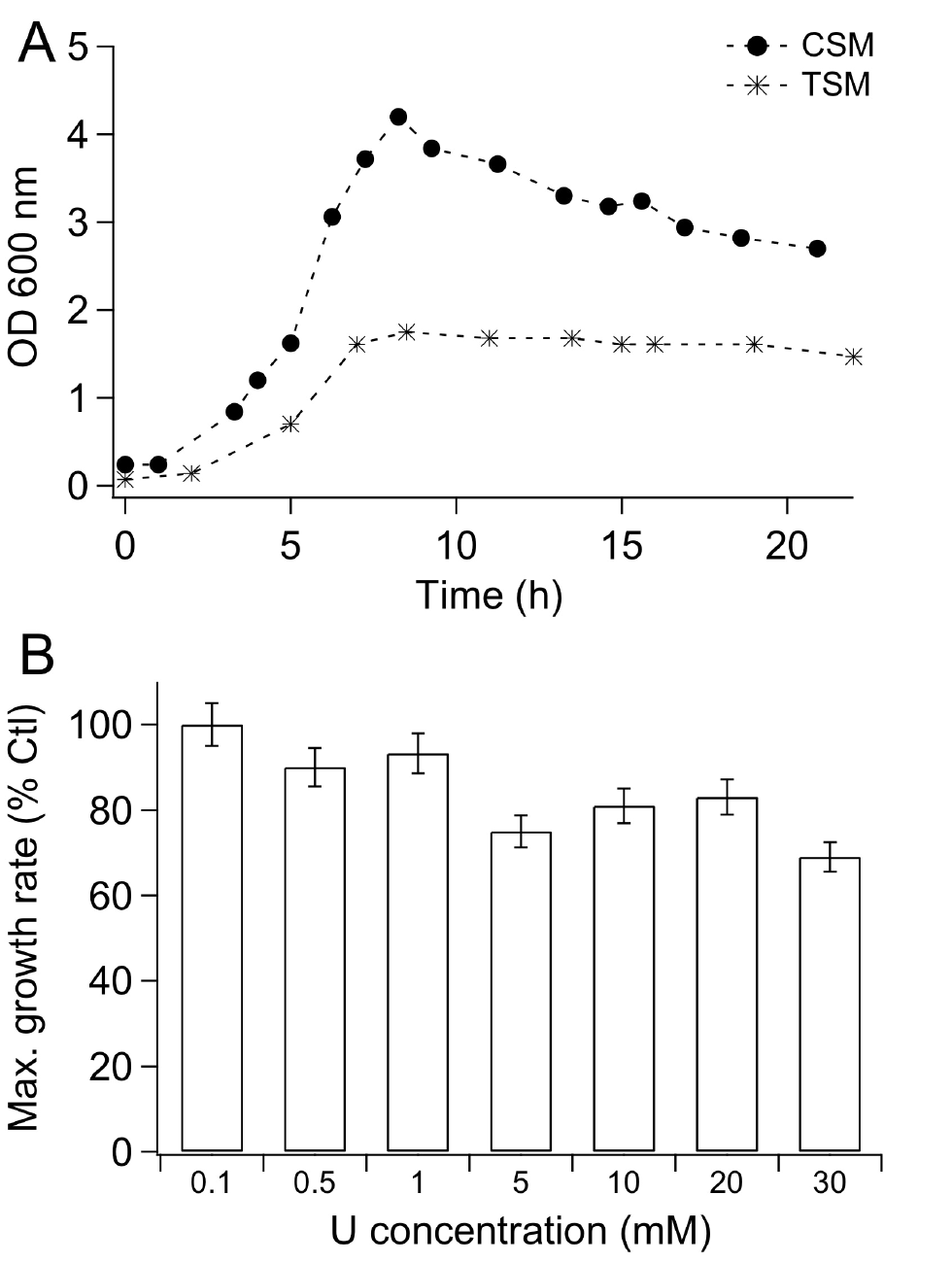

Supplement: Figure S1 — Growth of C. metallidurans CH34 in CSM vs. TSM medium. C. metallidurans classical growth medium is TSM; in order to avoid U precipitation during exposure this growth medium was adapted, i.e. Tris buffer was replaced by citrate, leading to a growth medium denominated CSM. (TIF) [file pone.0051783.s001.tif]

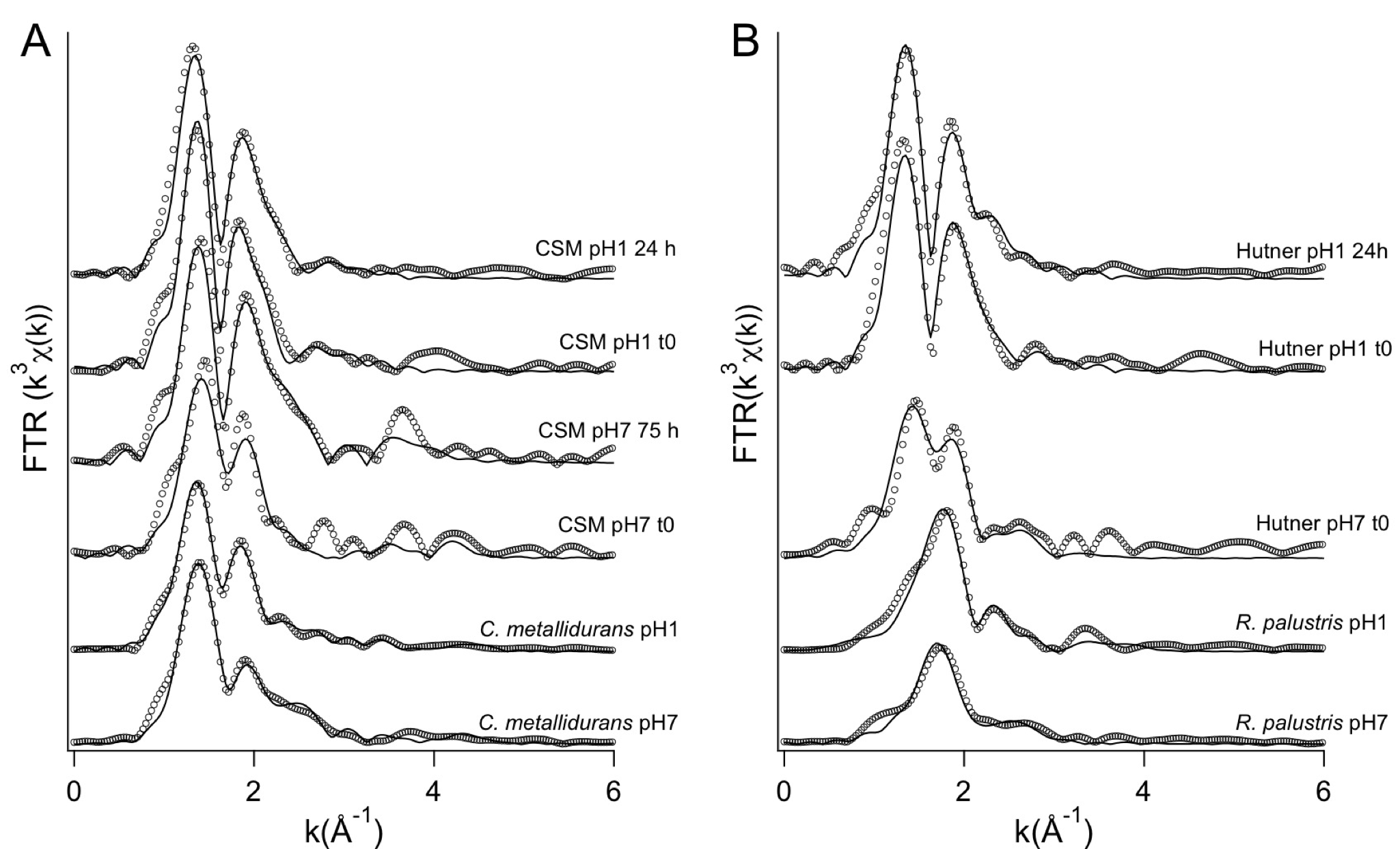

Supplement: Figure S2 — Fourier transforms of the EXAFS spectra recorded on exposure media and bacteria pellets. (TIF) [file pone.0051783.s002.tif]
